# Practical advice for selecting or determining trophic magnification factors for application under the European Union Water Framework Directive

**DOI:** 10.1002/ieam.4102

**Published:** 2018-11-26

**Authors:** Karen A Kidd, Lawrence P Burkhard, Marc Babut, Katrine Borgå, Derek CG Muir, Olivier Perceval, Heinz Ruedel, Kent Woodburn, Michelle R Embry

**Affiliations:** ^1^ McMaster University Hamilton Ontario Canada; ^2^ Mid‐Continent Ecology Division, National Health and Environmental Effects Laboratory US Environmental Protection Agency Duluth Minnesota; ^3^ RIVERLY Research Unit National Research Institute of Science and Technology for Environment and Agriculture (IRSTEA) Villeurbanne Cedex France; ^4^ Department of Biosciences University of Oslo Oslo Norway; ^5^ Environment & Climate Change Canada Burlington Ontario Canada; ^6^ French Agency for Biodiversity Vincennes France; ^7^ Fraunhofer Institute for Molecular Biology and Applied Ecology (Fraunhofer IME) Schmallenberg Germany; ^8^ Dow Chemical Midland Michigan USA; ^9^ Health and Environmental Sciences Institute Washington DC USA

**Keywords:** Bioaccumulation, Trophic magnification factor, Environmental Quality Standard, Water Framework Directive

## Abstract

European Union Directive 2013/39/EU, which amended and updated the Water Framework Directive (WFD; 2000/60/EC) and its daughter directive (2008/105/EC), sets Environmental Quality Standards for biota (EQS_biota_) for a number of bioaccumulative chemicals. These chemicals pose a threat to both aquatic wildlife and human health via the consumption of contaminated prey or the intake of contaminated food originating from the aquatic environment. EU member states will need to establish programs to monitor the concentration of 11 priority substances in biota and assess compliance against these new standards for the classification of surface water bodies. An EU‐wide guidance effectively addresses the implementation of EQS_biota_. Flexibility is allowed in the choice of target species used for monitoring to account for both diversity of habitats and aquatic community composition across Europe. According to that guidance, the consistency and comparability of monitoring data across member states should be enhanced by adjusting the data on biota contaminant concentrations to a standard trophic level by use of the appropriate trophic magnification factor (TMF), a metric of contaminant biomagnification through the food web. In this context, the selection of a TMF value for a given substance is a critical issue, because this field‐derived measure of trophic magnification can show variability related to the characteristics of ecosystems, the biology and ecology of organisms, the experimental design, and the statistical methods used for TMF calculation. This paper provides general practical advice and guidance for the selection or determination of TMFs for reliable application within the context of the WFD (i.e., adjustment of monitoring data and EQS derivation). Based on a series of quality attributes for TMFs, a decision tree is presented to help end users select a reasonable and relevant TMF. *Integr Environ Assess Manag* 2019;15:266–277. © 2018 The Authors. *Integrated Environmental Assessment and Management* published by Wiley Periodicals, Inc. on behalf of Society of Environmental Toxicology & Chemistry (SETAC)

## Key Points

- The EU Water Framework Directive requires the monitoring of priority substances in biota to assess compliance with Environmental Quality Standards (EQS_biota_).
- For comparisons to the EQS_biota_, concentrations of substances are normalized to a common trophic level by use of field‐derived trophic magnification factors (TMFs).
- However, TMF values for a chemical can vary among ecosystems.
- Herein, a decision tree is described and examples given to select an appropriate TMF, considering key aspects such as habitat type, experimental design, and appropriate data normalization.

## INTRODUCTION

One of the 2 main goals of the Water Framework Directive (WFD) adopted in 2000 by the Member States of the European Union is to achieve good “chemical status” by ensuring that the concentrations of chemicals of concern, or priority substances, are kept below their respective Environmental Quality Standards (EQSs) (European Parliament and European Commission [Link]). An EQS is defined as the concentration of a particular pollutant or group of pollutants in water, sediment, or biota that should not be exceeded in order to protect human health and the environment. EQSs were initially set for water only, except for hexachlorobenzene (HCB), hexachlorobutadiene (HCBD), and Hg, which were for biota (European Commission 2008). A revision of the priority substances list under the WFD occurred in 2013, leading to the addition of 8 priority substances targeting biota—namely, polybromodiphenylethers (PBDEs), perfluorooctane sulfonate (PFOS), hexabromocyclododecane (HBCDD; sum of 3 isomers), dioxins and dioxin‐like compounds, heptachlor and its epoxide, fluoranthene, other polycyclicaromatic hydrocarbons (PAHs; with benzo[a]pyrene as a marker for 5‐ and 6‐ring PAHs), and dicofol (European Parliament and European Commission 2013).

**Figure 1:**
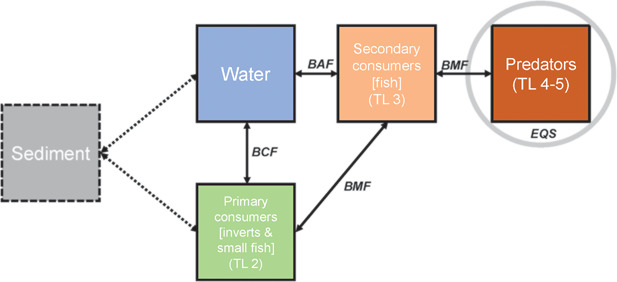
Theoretical food web used for deriving EQSs for biota. BAF = bioaccumulation factor; BCF = bioconcentration factor; BMF = biomagnification factor; EQS = Environmental Quality Standard; TL = trophic level.

Biota EQSs essentially refer to fish (with the notable exception of 5‐ to 6‐ring PAHs and fluoranthene, in which reference is made to crustaceans and mollusks) and should be applied to prey species occupying a trophic level (TL) that is sufficiently high in the food web to ensure the protection of top aquatic predators (assuming 100% reliance on that particular prey item). In general, for chemicals that are subject to biomagnification, the peak concentrations are attained in predators at TL 4 in freshwater food webs and at TL 5 for marine food webs, where the risk of secondary poisoning in top predators should be considered (see the simplified food web described in Figure 1, used for the determination of EQSs under the WFD).

In the application of the WFD and its “daughter directives” from 2008 and 2013, member states must provide periodic assessments of the chemical status of water bodies under their jurisdiction. As a consequence of the adoption of the 2013 directive (European Parliament and European Commission 2013), they will thus have to design and implement biota‐monitoring programs. With this perspective, the European Commission (2014) published a technical guidance document describing the general principles, rather than fixed standard rules, of such monitoring programs, for example, selection of sampling locations, design of sampling program, suitable matrix for chemical analysis, and data handling and compliance assessment. The selection of target fish species was kept flexible, considering the diversity of habitats and fish species distribution across Europe. According to this guidance (European Commission 2014), the comparability of data across member states within the European Union would be achieved by adjusting the biota‐monitoring results to a standard TL, that is, TL 4 for continental water bodies, and to a standard lipid content (5% or 0.05) or a standard dry weight (dw) content (26% or 0.26), by use of the following equations:

(1) $$\left[C_{adj-TL,norm}\right]=\left[C_{meas}\right]\times TMF^{\left(4-TL\left(x\right)\right)}\times 0.05/lipid$$

(2) $$\left[C_{adj-TL,norm}\right]=\left[C_{meas}\right]\times TMF^{\left(4-TL\left(x\right)\right)}\times 0.26/dw$$

where *C*_adj−TL,norm_ is the contaminant concentration adjusted to TL and lipid content (Equation (1)) (μg/kg‐lipid) or dry weight (Equation (2)) (μg/kg‐dw) basis, *C*_meas_ is the measured, nonnormalized contaminant concentration (μg/kg‐ww), and *TL*, *lipid*, and *dw* are the TL (based on expert knowledge, available databases, or stable isotope data [see below]), lipid content, and dry mass of the monitored species, respectively. The Equation (2) variant is proposed for contaminants for which accumulation is not influenced by the organism's lipid content, such as PFOS (Jones et al. 2003) or Hg (Visha et al. 2015).

TMF stands for “trophic magnification factor”; it represents the “diet‐weighted average biomagnification factor (BMF) of chemical residues across food webs” (Burkhard et al. 2013). In practice, the TMF is most often derived from the slope of the regression of log‐transformed, lipid‐adjusted chemical residues in food web organisms upon their corresponding TLs, calculated from stable N isotope (i.e., δ^15^N) data (Borgå et al. 2012). Over the past several decades, researchers have used δ^15^N of organisms as a proxy to assess their relative trophic position for inclusion in assessments of the trophic transfer of contaminants through marine and freshwater food webs (e.g., Broman et al. 1992; Kidd et al. 1995). The method was subsequently refined by calculating integer‐based TL values from δ^15^N with use of enrichment factors (i.e., increase in ^15^N from the diet or prey to the consumer, called Δ^15^N [Fisk et al. 2001]). The numeric TL for each species is calculated relative to a baseline species for that food web (e.g., mussels, midge, mayfly, zooplankton, etc.), which is assumed to occupy a base TL of 2.0. TL values for all other food web species (e.g., invertebrates and fish) are determined with the following equation:

(3) $${\text{TL}}_{\text{consumer}}=2.0+[\left(\delta^{15}N_{\text{consumer}}-\delta^{15}N_{\text{baseline}}\right)/\Delta^{15}N]$$

where TL_consumer_ is the TL of the organism, δ^15^N_consumer_ and δ^15^N_baseline_ are the δ^15^N data for an organism and the baseline species, respectively, 2.0 is the assumed TL of the baseline species, and Δ^15^N is the trophic enrichment factor (EF) constant for δ^15^N in the food web. For aquatic poikilothermic food webs, the trophic EF constant for δ^15^N (Δ^15^N) used to calculate TL values typically varies from 3.0‰ to more than 5.0‰ per TL step (DeNiro and Epstein 1981; Minagawa and Wada 1984; Post 2002; Jardine et al. 2006). A value of 3.4‰ per TL step has been recommended for constructing food webs without a priori knowledge of Δ^15^N or the ecology of the system (DeNiro and Epstein 1981; Minagawa and Wada 1984; Post 2002). Linear regressions of log‐transformed, lipid‐normalized biota concentrations versus TL are then used to determine TMF values, as shown below:

(4) $${\log10}_{}\left[C_{Lipid}\right]=a+b\times TL$$

where *C*_Lipid_ is the lipid‐normalized concentration of chemical, TL is the TL assigned to the species under analysis (Equation (3)), and *a* and *b* are the intercept and slope of the linear regression line, respectively. The slope *b* is then used to calculate TMF as

(5) $$TMF=10^{b}$$

or

(6) $$TMF=e^{b}$$

where *b* is the slope of the regression, with base 10 or *e* depending on the logarithmic transformation. If the regression slope for the chemical is based on δ^15^N instead of TL, then *b* is multiplied by an EF prior to calculating TMFs.

In the context of the WFD, TMF selection is therefore a critical issue, and reported values of this field‐derived metric may be quite variable for a given contaminant (Franklin 2016), even for well‐known and well‐studied chemicals like polychlorinated biphenyls (PCBs). The sources of TMF variability relate to the chemical properties, experimental design, and the ecosystem(s) considered. Furthermore, the use of TMFs on dissimilar systems or species also increases the overall uncertainty with respect to steady‐state assumptions, comparability of species included in the regression (Borgå et al. 2012), and the TL assignment used in both the TMF regression and for the monitored biota (Starrfelt et al. 2013).

While a few EU member states are considering developing specific sets of TMFs for the water bodies under their jurisdictions (especially for riverine ecosystems), others will use values obtained from the literature (e.g., peer‐reviewed publications, government reports). These numerical values will subsequently be used to adjust the body residues of a priority substance in monitored biota with a greater level of confidence to a common TL, in order to compare these adjusted measures with the corresponding EQSs. Generally, the goal is to minimize both Type I (false positives) and Type II (false negatives) errors, while recognizing that minimizing the Type II errors will be more protective of the environment. This purpose is somewhat different from that of assessments under the European Parliament and European Commission (2006) Registration, Evaluation, Authorisation, and Restriction of Chemicals (REACH) regulation or the Stockholm Convention on Persistent Organic Pollutants (2001) (POPs), which is to state whether or not biomagnification occurs, i.e., whether TMF is greater than 1 for the determination of the B criteria in persistent, bioaccumulative, and toxic (PBT) assessment under REACH (Conder et al. 2012).

Our objective is to provide general guidance for the selection of TMFs for reliable application within the context of the WFD (i.e., adjustment of monitoring data and comparison to the EQS). This document does not aim to provide a thorough review of TMF study design or individual studies themselves. Rather, it highlights recently published advances and developed approaches to evaluate and use TMFs that could help inform the implementation of biota EQSs under the WFD.

## SCIENTIFIC ISSUES RELATED TO USE AND APPLICATION OF TMFs

### Scope

For most of the priority substances for which a biota standard has been set, multiple field bioaccumulation studies are available. These data are essential for the following:

- To express the EQS_biota_ (usually for fish) as a concentration in another group of species considered suitable for environmental monitoring (e.g., mussels in the marine environment).
- To compare established EQS_biota_ values (usually for fish) with monitoring data from biota at different TLs, which requires adjustment of the levels of a priority substance. This adjustment allows for comparisons of contamination from different species and different sites to be made against the EQS.

The trophic magnification factors that should be used for this purpose are TMFs that refer to the pelagic food chain (i.e., water‐respiring organisms), excluding birds and mammals.

### Design and execution of TMF studies

Several metrics to describe the bioaccumulative capacity of compounds currently exist: *n*‐octanol/water partition coefficient [*K*_OW_], bioconcentration factor [BCF], bioaccumulation factor [BAF], biota sediment accumulation factor [BSAF], BMF, and TMF. Some are laboratory‐derived metrics based on ratios of measured concentrations (i.e., *K*_OW_, BCF) and global regulatory guidelines, while others are largely ratios of field‐determined measurements between abiotic exposure and levels accumulated in biota (i.e., BAF, BSAF). The metrics that explicitly account for biomagnification via dietary transfer (i.e., BMF and TMF) are ratios of the concentrations in predators over that of their prey or slope‐derived values from regressions of field concentrations (typically lipid‐based [see exceptions below], log normalized) versus TL, respectively. Biomagnification is a concern to risk assessors owing to the potential for elevated biotic concentrations causing adverse effects that may threaten the populations of higher TL species (Fisk et al. 2005; Gobas et al. 2009; Letcher et al. 2010). While techniques exist for the derivation of TMFs from food web contaminant data (Mackintosh et al. 2004; Borgå et al. 2012; Conder et al. 2012; ECETOC 2014), no standard guidance is currently present for global regulatory evaluation or conduct of TMF studies, although Conder et al. (2012) discuss the use of TMFs to assess bioaccumulation in a regulatory context.

The TMF approach assumes that diet is the major route of contaminant exposure and that relative TL is the main driver of their accumulation in organisms and food webs. The TMFs capture biomagnification processes occurring across food webs. However, other factors such as age, size, reproductive status, biotransformation efficiency, and omnivorous feeding affect contaminant residues in an organism and, if not properly addressed, may confound TMF determination. In addition, TMFs may be influenced by species phenology, migration, spatial variability in contaminant inputs, seasonal variability in contaminants of short‐lived species, and metabolism of the chemicals, some of which is described in additional detail in subsequent sections.

### Practical advice on choosing or determining a TMF

**Figure 2:**
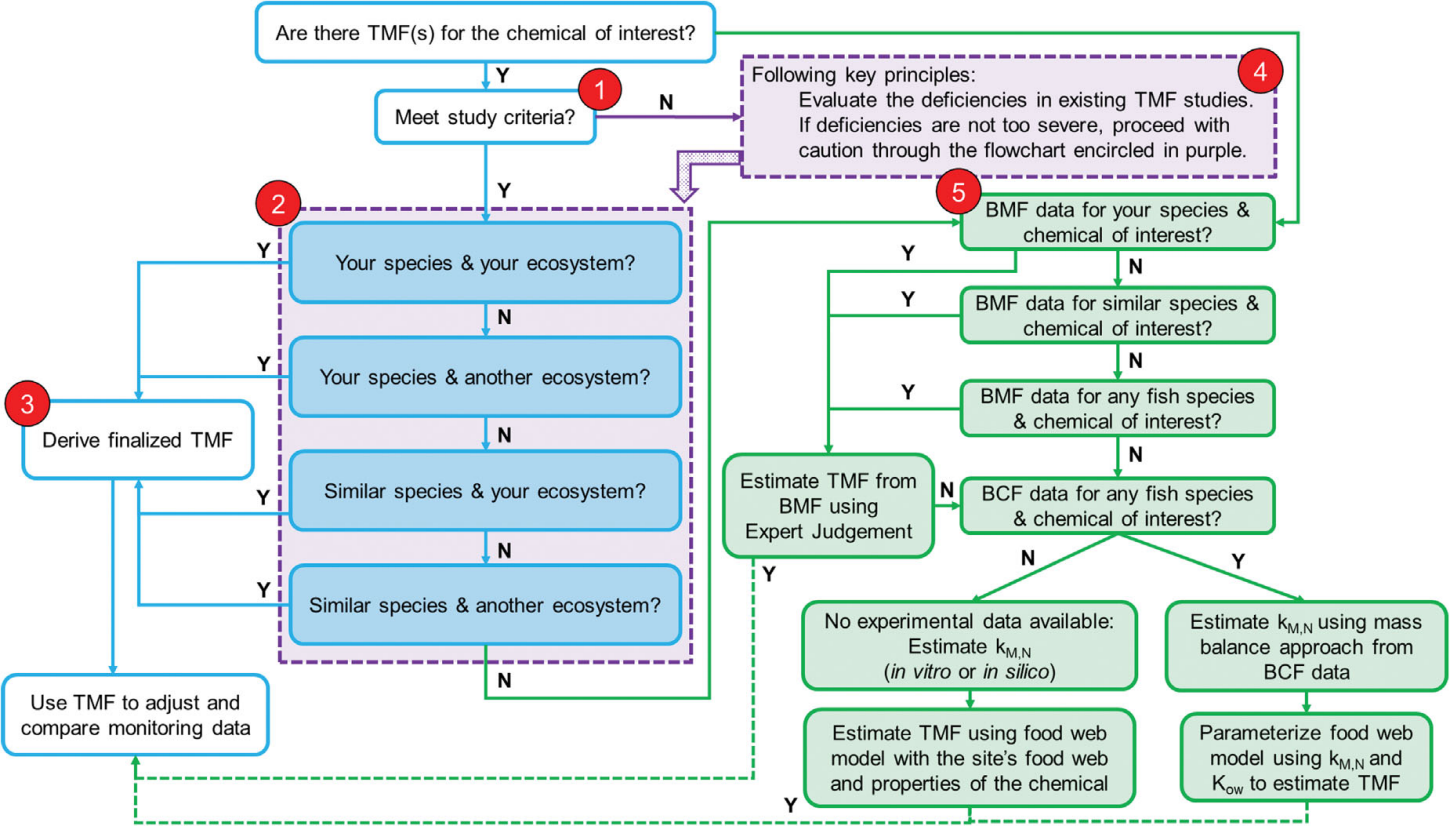
Flow chart describing general guidelines for evaluation of TMF studies. BCF = bioconcentration factor; BMF = biomagnification factor; k_M,N_ = biotransformation rate; *K*_OW_ = *n*‐octanol/water partition coefficient; N = no; TMF = trophic magnification factor; Y = yes.

This section provides guidance on how to select and apply a TMF that is most relevant to specific water bodies. The process is also identified as a series of numbered steps in Figure 2 and referenced in the appropriate sections.

TMFs are calculated by use of the antilog of the slope of the regression between log‐ or ln‐transformed contaminant concentrations versus TL, calculated from δ^15^N (in per mil, ‰), of food web organisms. TMFs and their underlying regressions can be found in the peer‐reviewed literature, although, as described below, some published studies will be more appropriate to use than others.

The following are brief guidelines for choosing a TMF to ensure that it is best suited to a specific site. These considerations are strongly recommended as they provide a higher level of confidence in the TMF values and their applicability. These study criteria are based upon several available reviews of the TMF literature for organics and Hg (Borgå et al. 2012; Conder et al. 2012; Lavoie et al. 2013; ECETOC 2014; Walters et al. 2016) and are outlined below.

#### Study criteria (Figure 2, Section 1)

- A minimum TL range of 2.0 (i.e., TL 2.0–4.0) in which several species are analyzed for the contaminant and δ^15^N. TL range is determined from the following:
  ‐ The difference in reported TLs for the highest‐TL fish species and lowest‐TL nonvertebrate taxa, or
  ‐ Dividing the difference in reported δ^15^N values for the highest‐TL fish species and lowest‐TL nonvertebrate taxa by the δ^15^N EF (Δ^15^N). A Δ^15^N value may range from 3.0‰ to 5.0‰ per TL but is typically assumed to be 3.4‰ for this calculation (DeNiro and Epstein 1981; Minagawa and Wada 1984; Post 2002).

- Fish whole‐body residue measurements.
  ‐ Whole‐body is preferred to analyses in fish muscle; otherwise conversion factors (or equations obtained from regressions) taken from the literature could be applied to estimate whole‐body burdens from muscle concentrations. Such an approach is nevertheless species and compound specific, and as such, not always applicable.

- Appropriate normalization (e.g., lipid, dry weight); see discussion on influence of normalization example below.
- Inclusion of several lower‐trophic‐level, nonvertebrate taxa (i.e. zooplankton, several different benthic invertebrate families).
- Reasonable balance with respect to sample numbers of lower‐ versus higher‐trophic‐level organisms.
- Adequate and balanced representation of samples for each TL.
  ‐ Differences in sample size among different levels of the food web can produce an “unbalanced” sampling design that requires the application of appropriate statistical methods to determine the TMF (Borgå et al. 2012; Powell et al. 2017, 2018). An unbalanced design may contribute to uncertainty in the TMF estimate.

- Measurements are on organisms that are known to be linked by diet through the food web.
  ‐ Assumptions are ideally supported through inspection of gut contents or δ^13^C or δ^34^S analyses (measure of energy sources supporting organisms) (Hecky and Hesslein 1995; Croisetiere et al. 2009) and are from the same habitat type (e.g., pelagic versus benthic) (Nfon et al. 2008).

- δ^15^N to δ^13^C stable isotope ratio data available and appropriate baseline organism used (Equation (3)).
- Measured contaminant concentrations in all biota samples are above the detection limit and appropriate analytical quality data are reported.
- All organisms collected within an appropriate or similar sampling period (e.g., 1 season).

#### Tiering in order of preference for the TMF (Figure 2, Section 2)

- Similar ecosystem: Appraisal based on physical (e.g., river vs. lake; latitude, mean annual temperature), chemical (nutrients, pH, etc.), and biological (food web structure) characteristics to the site of interest.
- Similar fish species: Appraisal based on ecological traits (e.g., feeding behavior) and biological (e.g., reproduction period) characteristics compared to targeted species at the site of interest.

#### Derivation of the TMF (Figure 2, Section 3)

In instances in which there are several relevant TMFs available for the chemical that meet the above recommendations, a geometric mean of those values should be calculated and applied. In cases in which these criteria cannot be met, the flow chart in Figure 2 provides some options for the next best choices for TMF values.

In addition, when studies do not present TMF values calculated as in Equations (5) or (6), it is possible to calculate them as follows (a δ^15^N difference of 3.4 ‰ per TL is assumed; see Post [2002]):

(7) $$\log\;(or\;\ln)\;conta\,\min ant\;concentrations=a+b\times \delta^{15}N$$

(8) $$TMF\;for\;\log\;conta\,\min ant\;concentrations=10^{b\times 3.4}$$

(9) $$TMF\;for\;\ln\;conta\,\min ant\;concentrations=e^{b\times 3.4}$$

These regressions can be based on either contaminant concentrations expressed on a wet weight or dry weight basis as the slopes do not change markedly upon conversion from one to the other (see below).

Once a TMF has been calculated, data for the monitored and analyzed species can be adjusted to TL 4 (for freshwaters) or TL 5 (for marine) if needed using Equation (1) or (2).

#### Evaluation of deficiencies in criteria for the measured TMF (Figure 2, Section 4)

When a TMF field study deviates from the criteria for a high‐quality study (defined above), the resulting TMFs will have additional uncertainties. The amount of additional uncertainty will depend upon the type and number of deficiencies in the study. Evaluations of the deficiencies should be performed on a case‐by‐case basis according to the following guidance:

- If fish fillets were analyzed, convert to whole‐body residue and then perform the regression again to calculate the TMF. If whole‐body conversion is not possible, lipid normalization for fillet data should be performed for nonionic organic chemicals.
- If organisms do not belong to the same food chain (e.g., based on gut content inspection or on δ^13^C or δ^34^S analysis), do not proceed.
- If the TL range is too small (e.g., less than 2.0 as per the Study Criteria above), the case requires expert judgment as to whether to use the TMF or not.
- If there is a limited number of lower‐trophic‐level invertebrates or insufficient replication, the case requires expert judgment as to whether to use the TMF or not.
- If the study includes endotherms, recalculate TMF excluding endotherms.
- If there is unbalanced sampling, recalculate TMF by using advanced statistical modeling such as a general linear mixed model (Bolker et al. 2009).
- If some concentration data are below the detection limit, the case requires expert statistical judgment.

#### Instances when there is not an appropriate TMF (Figure 2, Section 5)

In cases in which no TMF is available for the chemical in question, TMFs may be approximated from other measures of bioaccumulation. In Figure 2, Section 5, a series of approximations in descending order of their preference is provided. The first approximation assumes the TMF equals the BMF. This assumption is reasonable because TMF is a diet‐weighted average BMF of chemical residues across the food web (Burkhard et al. 2013). The BMF needs to be reflective of 1 TL step, and high‐quality BMFs can be measured in the laboratory with dietary exposure studies (OECD 2012). BMFs may be derived from field measurement, but they will have added uncertainty because the exact feeding behavior of the predator species is not easily determined. In Figure 2, Section 5, preference is given to BMFs available for the species of interest. If unavailable, BMFs available for a similar species are preferred over BMFs from any other fish species. Clearly, uncertainty increases as one drops through the tiers.

When high‐quality BMFs are unavailable, the second approximation is to use BCF data for fish. When BCF data are available, derive the fish's whole‐organism biotransformation rate (k_M,N_) for the chemical of interest by using a chemical mass‐balance model for fish (Arnot et al. 2008a, 2008b). Then, with a food web model (Arnot and Gobas 2004), estimate the TMF by using biota concentration data generated with the derived k_M,N_, the *K*_OW_ of the chemical, and a food web matching the ecosystem of interest.

When no bioaccumulation measurements exist, a third approximation is to estimate the TMF from biota data generated with an estimated biotransformation rate (k_M,N_) of the chemical in fish, the *K*_OW_ of the chemical, and a food web model with inputs matching the ecosystem of interest. Estimation of the k_M,N_ can be performed using in vitro and/or in silico techniques (Arnot et al. 2008a, 2008b).

Expert judgment is required when approximating TMFs with these methods (i.e., Figure 2, Section 5) because uncertainties in the approximated TMFs will increase as one proceeds from BMFs to BCFs to no experimental data. When an estimated TMF is derived, considerable effort should be expended to evaluate the reasonableness of the estimate.

## EXAMPLES

The following are selected brief illustrative case study examples of how the above‐mentioned considerations and situations can affect TMF values.

### Normalization

Owing to the heterogeneous distribution of chemicals in organisms, an adjustment may be required. For example, nonpolar compounds tend to bioaccumulate in lipid‐rich organisms (and in lipid‐rich tissues). An appropriate normalization considers the different lipid fractions of the different food web organisms. In other cases, the bioaccumulation may be related to the tissues’ protein content (e.g., PFOS, Hg). In these cases, the protein content (or %N as an approximation) may be used for normalizations (FAO 2003).

#### Dry weight normalization (Hg)

The influence of dry weight normalization on TMF values can be illustrated with a study on methylmercury (MeHg) by Clayden et al. (2013). The authors measured dry weight MeHg in pooled samples of several invertebrate taxa and total Hg (THg) in individuals of different fish species (for which total Hg is approximately MeHg; see Bloom [1992]) in several lake food webs. While they used individual data for the log Hg versus TL regressions, only average data per species could be used herein, but the regression slopes were similar to those originally published. The following percentage water values from the literature were applied: fish, 74% (European Commission 2014); zooplankton, 90% (Ovie and Ovie 2006); chironomidae, 79% (Frouz and Matěna 2015); and other invertebrates, 80% (Leeves 2011). If the water content of all aquatic species were equal, no difference between wet and dry weight–based TMFs would be observed. For the tested data set, the wet weight–based TMFs were about 15% higher than the dry weight–normalized TMFs (Table S1). However, as the range of reported MeHg TMF is relatively large (e.g., mean ± standard deviation TMF = 8.3 ± 7.5 based on a metaanalysis of wet weight data from 101 freshwater datasets [Lavoie et al. 2013]), the differences observed between wet and dry weight normalization are less significant. In addition, Wyn et al. (2009) compared slopes of the regressions of log MeHg and THg concentrations on a wet versus dry weight basis against δ15N in lake food webs and found no significant differences within lakes. In this case study, normalization of MeHg tissue concentrations to dry weight may be more meaningful than using wet weight data because MeHg is associated with cell proteins and dry weight is likely more strongly correlated with tissue protein content.

#### Lipid normalization

Lipid weight (lw) normalization has been used for the calculation of TMFs for nonionic organic chemicals (Borgå et al. 2012) but not for ionizable chemicals such as PFOS and MeHg. There may be situations in which it might be useful to compare TMFs on the same basis, i.e., using wet (or dry) weight TMFs. Houde et al. (2008b) investigated this comparison for lake trout food webs, where TMFs for 2 PCB congeners (PCB153, a hexachlorobiphenyl, and PCB52, a tetrachlorobiphenyl) were calculated for 17 lake trout food webs with the wet weight (ww), whole‐body data (Table S2). Wet weight–based TMFs for both congeners were consistently higher than those based on lipid weight (i.e., 1.64‐ and 1.61‐fold higher for PCB153 and PCB52, respectively). This result is due to the general increase in percentage lipid with TL in these food webs. Typically, lake trout, the top predator in all the lakes in the study, had the highest percentage lipid (∼5%–15%), while zooplankton had the lowest (∼0.5%–2%). Houde et al. (2008b) reported that wet weight TMFs for PCB153 and PCB52 were weakly positively correlated with lake area and maximum depth but not with aqueous dissolved organic C or food chain length (Table S3). However, given the importance of lipid as a covariate for these congeners, it would seem appropriate to investigate such relationships with only lipid‐normalized TMFs. Thus, reporting concentrations on a lipid‐normalized basis removes the effect of lipid content on PCB accumulation and allows the identification of trophic magnification itself.

### Whole‐body measurements in fish: The example of PBDEs

Ideally, TMFs should be based on whole‐body concentrations measured in all organisms of the food web (Borgå et al. 2012). Nevertheless, for practical or ethical (e.g., nonlethal sampling) reasons, contaminants in higher TL species are often analyzed in specific organs or tissues, such as the dorsal muscle in fish. This practice could lead to biased TMF estimates. PBDEs partition to lipids and are used in the case study below. The consequences of using specific tissues instead of whole‐body measurements would be different for ionizable and protein‐binding organic chemicals.

Among the 14 studies with TMFs for PBDEs (Table S4), 2 analyzed PBDEs in whole‐body homogenates and the remaining used dorsal muscle samples or whole‐body concentrations for small fish and fillet concentrations for larger individuals or species. As PBDE concentrations are higher in the liver or the perivisceral adipose tissues than in dorsal muscle for a wide array of freshwater and marine species (Burreau et al. 2000; Voorspoels et al. 2003; Kim et al. 2015), TMFs for PBDEs could be underestimated when based on fillet measurements in fish. The few available fillet to whole‐body concentration ratios are about 3 (range, 2.6–4.9). TMFs derived with residues from differing tissues across the food web will have added uncertainty over those based upon whole‐body residues. For nonpolar organics, adjustments of residues from fillets and specific organs to whole‐body by adjusting for lipid content is often reasonably sufficient for deriving a valid TMF.

### Sampling design effect

One of the most challenging and important aspects of a field study for TMFs is the sampling of the aquatic food web. To adequately characterize the food web, sufficient numbers of key organisms from each TL must be obtained. Individual samples of tissues from higher TLs (e.g., piscivorous fish) are generally much more easily collected than lower‐trophic‐level organisms, such as pelagic or benthic invertebrate species. As a result, data sets used for regression analysis are usually heavily weighted with samples of higher TL species. In extreme cases of unbalanced designs, TMF values derived from these data sets can be more reflective of biomagnification among these higher TLs rather than through the complete food web. For example, in the Paguchi Lake (Canada) food web, the TMF for PCB153 was mainly influenced by the lipid‐adjusted concentrations in lake trout (*Salvelinus namaycush*), which represented approximately 50% of the samples (Houde et al. 2008a). In contrast, Poma et al. (2014) examined TMFs (and BMFs) of brominated flame retardants in the pelagic food web of Lake Maggiore and included 7 pooled samples for each of 2 fish species (sampled over 4 seasons) and 12 bulk zooplankton samples (collected at 3 sites and over 4 seasons).

For most studies, organisms in upper TLs are typically analyzed individually for stable isotopes and lipid‐adjusted chemical concentrations because this practice provides information on variability of individual organisms. For lower‐TL species, it is often necessary to pool or composite samples to provide an adequate mass for analysis; these pooled samples would be more representative of the population than separate individuals. If composite samples are required, it is recommended that multiple composite samples be collected in the field and analyzed as separate samples. Multiple composite samples create a problem for food webs where there is low species diversity because regression slopes can be driven by the “end” species when N is low. Lower‐TL organisms (i.e., zooplankton or benthic invertebrates) are typically feeding over a smaller area than higher TL organisms, so sampling location may also influence chemical concentrations in these organisms, as shown for PCBs in the Detroit River (McLeod et al. 2014). By comparison, top predators can act as ecological integrators (McCann et al. 2005) by consuming prey over large areas relative to more localized dietary items. Fish migration and spatial heterogeneity in contaminant concentrations (gradients or patchiness) were shown to be important factors influencing the magnitude and variation of TMFs (Kim et al. 2016).

Borgå et al. (2012) and Kim et al. (2016) found that most study designs having 30–40 samples would have only been able to detect (log) lipid‐adjusted concentration versus TL regression slopes with an absolute value greater than 0.3 to 0.5 (i.e., TMF values greater than 2.0–3.2) as being statistically different from a regression slope of 0 (i.e., TMF = 1.0). Such study designs are therefore unlikely to detect significant regression slopes for contaminants with apparent TMF values in the range of 0.5 to 2.0. Borgå et al. (2012) found that, with the level of variability associated with past experimental designs, only very large sampling sizes (N, 60–100) could detect regression slopes that were different from 0 (TMFs in the range of approximately 1.5 to 2.0). In addition, variability in concentration and/or TL assignment also significantly affects sampling design. For example, statistically significant regression slopes for contaminants having apparent TMFs as low as 1.4 to 1.6 can be detected with sample sizes of 20–30 if low variability is associated with the regression (Borgå et al. 2012).

### Sufficient knowledge of the food web (chloroalkanes, PFOS)

PFOS is a WFD priority substance for which an EQS has been established because there is considerable evidence that it biomagnifies (reviewed by Franklin [2016] and Houde et al. [2011]). TMFs for PFOS in aquatic food webs that include fish and no endotherms range widely from less than 1 to 6.4 (Table S5), making it difficult to choose a specific value. The TMFs are also very dependent on the feeding relationships of the organisms. For example, Martin et al. (2004) reported a TMF of 5.9 for the Lake Ontario pelagic food web (*Mysis*, alewife, rainbow smelt, lake trout) and estimated (on the basis of 2 TLs) a TMF of 1.86 for a benthic food web of *Diporeia* and sculpin. Houde et al. (2008a) reported a TMF for PFOS of 3.8 for the combined benthic–pelagic food web. Li et al. (2008) observed a strong relationship of log PFOS vs. TL (5 species) in Gaobeidian Lake in Beijing after tilapia, a benthic feeder, was omitted from the regression. In contrast to almost all other studies with PFOS, Lescord et al. (2015) found TMFs of less than 1 for the benthic‐based food webs of landlocked Arctic char (chironomids, juvenile char, adult char). They attributed this result to the benthic feeding, which was the predominant dietary pathway for adult char.

Most studies of PFOS in aquatic food webs have used whole‐body concentrations for fish (Table S5). The low TMFs found by Lescord et al. (2015) may be due to measurements of PFOS in fish muscle rather than whole‐body homogenates. Similarly, Houde et al. (2006) estimated whole‐body concentrations of PFOS from concentrations in plasma of live dolphins or liver tissues of dead animals and found that the resultant TMFs were lower by a factor 1.8 to 5.6 (depending on the location and extent of the food web considered) owing to lower whole‐body concentrations of this priority substance.

Almost all PFOS trophic magnification studies have reported results on a wet weight basis. An exception is Zhou et al. (2012), who used dry weight concentrations to calculate BAFs but not TMFs. As noted for MeHg, it is likely that TMFs would be lower for PFOS on a dry rather than a wet weight basis owing to higher water content of phytoplankton and invertebrates than of fish. However, to the best of our knowledge, this assumption has not been investigated for PFOS.

Kelly et al. (2009) compared PFOS TMFs calculated with protein‐normalized concentrations (TMF_PW_) with the conventional wet weight (TMF_WW_) approach and found that TMF_PW_ was lower than TMF_WW_ (11 vs. 17.4) owing to differences in protein content of various food web organisms. However, their Arctic marine food web was composed of samples from multiple sources and not all organisms were necessarily linked by diet. A bioaccumulation model based on protein binding (serum albumin, fatty acid binding proteins, and organic anion transporters) successfully predicted concentrations of PFOS in fish tissues (Ng and Hungerbühler 2013, 2014). However, to our knowledge, protein normalization of PFOS tissue concentrations or protein content of organisms has not been routinely reported. Phospholipids have also been used in modeling of bioaccumulation of PFOS (Armitage et al. 2013; Ng and Hungerbühler 2014) owing to their observed strong interaction with phospholipid bilayers (Xie et al. 2010). However, it appears that phospholipid normalization of PFOS concentrations in food web studies has not been done.

In summary, a wide range of PFOS TMFs have been reported for aquatic food webs. However, the range can be narrowed considerably by careful consideration of study designs, especially that the organisms are energetically linked through the food web and from the same habitat type and that appropriate tissues are analyzed.

### Studies from “nonrelevant” systems or food webs (MeHg)

Although few reviews exist that assess how TMFs range between lentic and lotic systems or marine versus freshwater, it is possible to examine how this context affects TMFs for MeHg as global food web studies were compiled and contrasted (Lavoie et al. 2013). From Table  2 of the article by Lavoie et al. (2013), average TMFs differed across latitudinal classes but most markedly between tropical and polar systems for freshwaters (3.5 vs. 8.9; temperate average, 6.5) and between tropical and temperate marine food webs (2.9 vs. 7.6; polar, 5.1). Similarly, food webs of rivers had a higher average TMF (8.2) than that of lakes (6.2). Finally, system productivity also appears to affect TMF values, with averages of 3.5, 7.6, 6.5, and 6.5 for hypereutrophic, eutrophic, mesotrophic, and oligotrophic systems, respectively. It should be noted that these values represent studies with diverse sampling strategies and species compositions and that limited studies were available for some system types. Despite these caveats, these results suggest that the use of a TMF value from a system with different physical or chemical characteristics will result in skewed calculations of chemical burden and associated comparisons to EQS_biota_.

### Data‐poor chemicals (dicofol)

Dicofol is an organochlorine pesticide consisting of 2 isomers (*p,p*′‐dicofol and *o,p*′‐dicofol) and is used as a miticidal pesticide and acaricide on fruits, vegetables, ornamentals, field crops, cotton, Christmas tree plantations, and nonagricultural outdoor buildings and structures (UNEP/POPS 2015). Dicofol log *K*_OW_ values reported in the literature range from 3.5 to 6.06 (UNEP/POPS 2015), with laboratory measured log BCF values ranging from 3.49 to 4.32 (Table S6).

Additionally, dicofol residues have been reported in numerous environmental media, including fish (da Silva et al. 2016), birds (Malik et al. 2011; Luzardo et al. 2014), and milk (USEPA 2009). There are no studies reporting BMFs or TMFs for dicofol (UNEP/POPS 2015).

In following the general guidance in Figure 2, one would proceed to Section 5 owing to the lack of TMF data for dicofol and then to the “BCF data for any fish species and chemical of interest?” box, because there are BCF data for carp and fathead minnow (Table S6). For the carp data, Arnot et al. (2008b) estimated dicofol whole‐fish biotransformation rates to range from 0.0062 to 0.0083 (d^−1^), using a mass‐balance approach with a log *K*_OW_ of 5.02. To estimate the TMF, a food web consisting of sediment, phytoplankton, zooplankton, forage fish, and piscivorous fish was set up and the TMF was estimated with AQUAWEB (Arnot and Gobas 2004) (see Table S7 for food web inputs). A TMF value of 0.75 was estimated with the AQUAWEB for piscivorous fish, and the TL of the piscivorous fish was estimated to be 4.0 on the basis of the diets of the organisms within the food web.

### Chemicals that are metabolized

Biotransformation has long been recognized as an important source of variation in predictions of bioaccumulation. Recent attempts to evaluate the relative importance of chemical, ecological, biological, and environmental factors in determining TMFs show that differences in biotransformation rates among hydrophobic organic chemicals could explain a significant amount of TMF variation (Kim et al. 2016; Walters et al. 2016). Typically, chemicals with high metabolic biotransformation rates are less likely to biomagnify in higher‐level organisms even if they exhibit high *K*_OW_ values (i.e., *K*_OW_ ≥ 10^5^). Conversely, slowly biotransformed organic chemicals are typically biomagnified. A biotransformation rate (k_M,N_) of approximately 0.025 d^−1^, representing a loss of 2.5% of the chemical in the organism per day, is sufficient to prevent trophic magnification for most substances (Kim et al. 2016).

In a metaanalysis, Walters et al. (2016) analyzed more than 1500 TMFs to identify organic chemicals predisposed to biomagnifying in aquatic food webs. For the 27 PAHs included in that study, TMF values ranged from 0.11 to 1.2, with a median value of 0.46 (geometric mean = 0.45). TMFs of PAHs identified as priority or priority hazardous substances under the WFD are shown in Table S8. Study quality criteria are generally met regarding the minimum TL range of 2.0 and the appropriateness of the methods used for the normalization of PAH tissue concentrations. In the study by Brisebois (2013), however, a large proportion of the concentrations were below the limits of detection (i.e., 70% of the samples), indicating a greater uncertainty associated with the TMF. For both benzo[a]pyrene and fluoranthene, reported TMF values were consistently less than 1, except for those listed by Wang et al. (2012). In this latter study, however, TMFs were derived from nonsignificant regressions and did not include lower‐trophic‐level nonvertebrate taxa. Some food web studies listed in Table S8 include endotherms, potentially affecting the value of TMFs because these food webs tend to be longer, increasing the likelihood of detecting a significant positive (or negative) slope in the chemical residue versus TL relationship. Recalculation of the TMFs obtained by Wan et al. (2007), either including or excluding birds from the data set, indicated no significant differences in slopes of the resulting regression lines (−0.52 vs. −0.61), yielding similar TMF values for total PAHs (i.e., 0.30 and 0.24, respectively).

For most studies identified in Table S8, PAH concentrations were measured in fish muscle, where PAHs generally do not accumulate (unlike what is observed in the bile or the liver [Zhao et al. 2014]) and may then not be representative of the “true” ambient PAH exposure. In fish, as in other vertebrates, biotransformation enzymes convert lipophilic organic chemicals into more water‐soluble metabolites, mainly in the liver, which are mainly excreted via bile in the feces (Strobel et al. 2015). In order to assess exposure of fish to PAHs, some authors therefore recommend determining PAH metabolite levels in the bile (Van der Oost et al. [2003] and literature cited herein).

In the case of metabolizable chemicals that undergo trophic dilution through the food web, the use of TMF values for the adjustment of monitoring data is not advisable. Instead, as concentrations of such chemicals generally peak in organisms occupying lower trophic levels, their EQSs should be applicable in lower‐TL taxa such as bivalves and crustaceans.

## SUMMARY AND CONCLUSIONS

A decision tree for selecting the most appropriate TMF for use within the EU WFD has been proposed herein. The TMFs are used to adjust chemical monitoring data for fish to the species of interest, that is, species consumed by humans and/or preyed upon by birds and mammals, to protect environmental and human health. The decision tree provides a hierarchical process starting with high‐quality TMF measurement studies and proceeds down through methods with increasing uncertainty. When there are no measured TMFs for the chemical of interest, the decision tree drops to estimating TMFs from BMF data or deriving TMFs with estimated or measured biotransformation rates for the chemical combined with a site‐specific food web model. Application of the decision tree requires best professional judgment, especially in cases in which a TMF is not available for the species of interest (or similar species) or a similar system. In developing the decision tree and the related case studies, we observed that many field studies were not designed to specifically measure TMFs and often do not meet the high‐quality criteria provided in this manuscript. This decision tree, we believe, will enable EU member states to better implement their monitoring programs for priority substances in biota and assess compliance against the EU's new standards for the classification of surface water bodies.

The decision tree proposed herein requires best professional or expert judgment at various decision points. There are currently no formalized, agreed‐upon methods, for example, no OECD methods, for determining TMFs, and providing defined criteria for these decision points requires further studies. In this paper, the decision tree and criteria set forth for high‐quality TMF studies can support the refinement of methods to conduct field studies for measuring TMFs. TMFs generated with such a formalized and standardized method would reduce the need for expert judgment in a broader regulatory context such as the Water Framework Directive.

## Disclaimer

The authors declare no conflicts of interest. The views expressed in this article are those of the authors and do not necessarily represent the views or policies of the US Environmental Protection Agency, Environment and Climate Change Canada, or the French Agency for Biodiversity.

## SUPPLEMENTAL DATA

**Table S1.** Exemplary comparison of MeHg TMF, calculated with data from the study by Clayden et al. (2013) on wet and dry weight basis.

**Table S2.** Comparison of TMFs for CB153 and CB52 calculated with lipid‐normalized concentrations (from Houde et al. 2008b) with TMF based on wet weight from the same food webs.

**Table S3.** Correlation analysis of wet weight‐based TMFs for CB153 and CB52 with lake characteristics.

**Table S4.** PBDE studies.

**Table S5.** TMF values for PFOS in aquatic food webs with fish as the top predator.

**Table S6.** Laboratory measured BCF values for dicofol.

**Table S7.** Input parameters and conditions for the Arnot–Gobas AQUAWEBv1.2 model.

**Table S8.** TMF values commonly reported for PAHs identified as PS/PHS under the WFD, and main attributes of aquatic food webs from which they are derived.

## Supporting information

This article includes online‐only Supplemental Data.

Supporting Data S1.

## Data Availability

Supplemental files have been made available that pertain to discussions within this paper.
