# Supplementary material for: Practical advice for selecting or determining trophic magnification factors for application under the European Union Water Framework Directive
Source: Integr Environ Assess Manag. 2018 Nov 26;15(2):266–77. doi: 10.1002/ieam.4102 (PMC6719707; doi:10.1002/ieam.4102)
Supplement: Supplementary file 1 — Supporting Data S1. [file IEAM-15-266-s001.docx]

**Practical Advice for Selecting or Determining Trophic Magnification Factors for**

**Application Under the European Union Water Framework Directive**

**SUPPLEMENTAL DATA**

Karen A Kidd,^†^ Lawrence P Burkhard,^‡^ Marc Babut,^§^ Katrine Borgå,^‖^ Derek CG Muir,^#^ Olivier Perceval,

^††^ Heinz Ruedel,^‡‡^ Kent Woodburn,^§§^ and Michelle R Embry^‖‖*^

^†^McMaster University, Hamilton, ON L8S 4L8, Canada; karenkidd@mcmaster.ca

^‡^Mid-Continent Ecology Division, National Health and Environmental Effects Laboratory, US Environmental Protection Agency, 6201 Congdon Blvd., Duluth, MN 55804, USA; burkhard.lawrence@epa.gov

^§^RIVERLY Research Unit, National Research Institute of Science and Technology for Environment and Agriculture (IRSTEA), 5 rue de la Doua CS 20244, 69625 Villeurbanne Cedex, France; marc.babut@irstea.fr

^‖^Department of Biosciences, University of Oslo, PO Box 1066 Blindern, N-0316 Oslo, Norway; katrine.borga@ibv.uio.no

^#^ Environment & Climate Change Canada, Burlington, ON L7S 1A1, Canada; derek.muir@canada.ca

^††^French Agency for Biodiversity, Site de Vincennes, Le Nadar, Hall C, 5 square Félix Nadar, 94300 Vincennes, France; olivier.perceval@afbiodiversite.fr

^‡‡^Fraunhofer Institute for Molecular Biology and Applied Ecology (Fraunhofer IME), Auf dem Aberg 1, 57392 Schmallenberg, Germany; heinz.ruedel@ime.fraunhofer.de

^§§^Dow Chemical, Midland, MI 48640, USA; kent.woodburn@dowcorning.com

^‖‖^Health and Environmental Sciences Institute, 740 15th Street NW, Sixth Floor, Washington, DC 20005, USA; membry@hesiglobal.org

*Address correspondence to Michelle R. Embry, Health and Environmental Sciences Institute, 740 15th Street NW, Sixth Floor, Washington, DC 20005, USA. Tel: +1 202-659-3306, ext. 132. Fax: +1 202-659-3617. E-mail: [membry@hesiglobal.org](mailto:membry@hesiglobal.org)

**Table S1.** Exemplary comparison of MeHg TMF calculated with data from Clayden et al. (2013) on wet and dry weight basis

| **Lake** | **Regression slope* in Clayden et al. (2013) (individual data, dw)** | **Regression slope calculated here  (aggregated data sets, dw)** | **TMF  dw-based (normalized)  (calculated from slope using modified equation e^1^)** | **Regression slope calculated here  (aggregated data sets, ww)** | **TMF  ww-based  (calculated from slope using modified equation e^1^)** |
| --- | --- | --- | --- | --- | --- |
| Big Dam West | 0.128 ± 0.010 | 0.109 ± 0.034 | 2.3 ± 1.3 | 0.125 ± 0.039 | 2.7 ± 1.4 |
| Big Dam East | 0.157 ± 0.007 | 0.154 ± 0.033 | 3.3 ± 1.3 | 0.170 ± 0.037 | 3.8 ± 1.3 |
| Beaverskin^§^ | 0.229 ± 0.009 | 0.229 ± 0.026 | 6.0 ± 1.2 | 0.249 ± 0.037 | 7.0 ± 1.3 |

Abbreviations: dw, dry weight; MeHg, methylmercury; THg, total Hg; TMF, trophic magnification factor; ww, wet weight.

*Regression slopes ± standard error of log Hg (THg for fish and MeHg for invertebrates; μg g^−1^ of dw) versus δ^15^N (‰).
^§^See also Wyn et al. (2009).

^1^Slopes based on δ^15^N values are multiplied by enrichment factors (herein we used 3.4‰) prior to using them in equation e.

**Table S2.** Comparison of TMFs for PCB153 and PCB52 calculated with lipid normalized concentrations (from Houde et al. 2008b) with TMF based on wet weight from the same food webs

| **Lake** | **Region** | **TMF** | **TMF** | **TMF** | **TMF** | **CB153** | **CB 52** |
| --- | --- | --- | --- | --- | --- | --- | --- |
|  |  | PCB 153 (lw) | PCB 52 lw | PCB 153 (ww) | PCB 52 (ww) | Ratio lw/ww | Ratio lw/ww |
| Athabasca | Northern | 4.9 ± 0.5 | 2.4 ± 0.7 | 6.0 ± 0.8 | 2.9 ± 1.0 | 0.82 | 0.82 |
| Champlain | Southern | 2.2 ± 0.2 | 1.6 ± 0.5 | 3.7 ± 0.2 | 2.7 ± 0.3 | 0.59 | 0.59 |
| Cold | Northern | 4.9 ± 0.7 | 1.7 ± 0.8 | 6.5 ± 0.9 | 2.2 ± 0.9 | 0.76 | 0.76 |
| Eva | Northwestern Ontario | 4.4 ± 0.4 | 1.8 ± 0.5 | 11 ± 0.7 | 4.6 ± 0.6 | 0.39 | 0.39 |
| Grist | Northern | 3.4 ± 0.3 | 2 ± 0.4 | 5.6 ± 0.4 | 3.2 ± 0.4 | 0.61 | 0.61 |
| Kingsmere | Northern | 2.3 ± 1.1 | 2.5 ± 0.7 | 2.5 ± 0.9 | 2.7 ± 0.7 | 0.93 | 0.93 |
| LaclaRonge | Northern | 3.8 ± 0.4 | 2.7 ± 0.8 | 4.5 ± 0.5 | 3.3 ± 0.9 | 0.84 | 0.84 |
| Namur | Northern | 2.3 ± 0.6 | 3.2 ± 0.6 | 5.2 ± 0.7 | 7.3 ± 0.9 | 0.44 | 0.44 |
| Opeongo | Southern | 2.8 ± 0.3 | 1.4 ± 0.5 | 5.3 ± 0.5 | 2.6 ± 0.4 | 0.54 | 0.54 |
| Paguchi | Northwestern Ontario | 3.4 ± 0.4 | 1.8 ± 0.5 | 7.3 ± 0.5 | 3.9 ± 0.5 | 0.46 | 0.46 |
| Reindeer | Northern | 3.7 ± 0.4 | 1.0 ± 1.4 | 3.6 ± 0.5 | 0.9 ± -1.1 | 1.01 | 1.14 |
| Sandybeach | Northwestern Ontario | 3.8 ± 0.3 | 1.8 ± 0.4 | 8.1 ± 0.6 | 3.8 ± 0.5 | 0.47 | 0.47 |
| Seneca | Southern | 3.5 ± 0.4 | 1.3 ± 1.0 | 6.4 ± 0.9 | 2.5 ± 1.0 | 0.54 | 0.54 |
| Simcoe | Southern | 1.5 ± 0.5 | 1.4 ± 0.4 | 4.3 ± 0.5 | 4.1 ± 0.4 | 0.34 | 0.33 |
| Superior | Southern | 6.0 ± 0.5 | 4.5 ± 0.4 | 10 ± 0.7 | 7.8 ± 0.7 | 0.58 | 0.58 |
| Thunder | Northwestern Ontario | 3.6 ± 0.5 | 1.8 ± 0.3 | 8.0 ± 1.2 | 4.0 ± 0.6 | 0.44 | 0.44 |
| Wollaston | Northern | 2.3 ± 0.6 | 2.5 ± 0.7 | 3.1 ± 0.7 | 3.3 ± 0.7 | 0.75 | 0.75 |
| Cayuga | Southern | 1.8 ± 1.6 | 1.2 ± 2.6 | 3.6 ± 1.3 | 2.3 ± 0.7 | 0.50 | 0.50 |
|  |  |  |  |  |  |  |  |
| Overall means | | 3.4 | 3.4 | 2.0 | 5.9 | 0.61 | 0.62 |
| Northern |  | 3.4 | 2.2 | 4.6 | 3.2 | 0.77 | 0.79 |
| Northwestern Ontario |  | 3.8 | 1.8 | 8.7 | 4.1 | 0.44 | 0.44 |
| Southern |  | 3.0 | 1.9 | 5.6 | 3.7 | 0.52 | 0.51 |

Wet weight concentrations are given in the supporting information for Houde et al. (2008b). TMFs were calculated using the individual data.

Abbreviations: lw, lipid weight; TMF, trophic magnification factor; ww, wet weight.

**Table S3.** Correlation analysis of wet weight–based TMFs for PCB153 and PCB52 with lake characteristics

| **Lake** |  | **z_max_ (m)** | **Area (ha)** | **DOC (mg/L)** | **Food web length** |
| --- | --- | --- | --- | --- | --- |
| Athabasca |  | 120 | 790000 | 7.4 | 9.3 |
| Champlain |  | 122 | 112700 | 3.7 | 11.9 |
| Cold |  | 99 | 37300 | 6.6 | 9.6 |
| Eva |  | 54 | 1708 | 5.8 | 8.5 |
| Grist |  | 43 | 2500 | 7.5 | 8.0 |
| Kingsmere |  | 47 | 4700 | 6.6 | 7.4 |
| LaRonge |  | 38 | 117800 | 7.1 | 7.9 |
| Namur |  | 27 | 4200 | 8.6 | 6.2 |
| Opeongo |  | 52 | 5860 | 6.1 | 7.0 |
| Paguchi |  | 54 | 2448 | 6.3 | 8.6 |
| Reindeer |  | 215 | 556900 | 5.7 | 8.6 |
| SandyBeach |  | 41 | 3820 | 8.1 | 8.7 |
| Seneca |  | 193 | 17500 | 4.4 | 5.6 |
| Simcoe |  | 41 | 72500 | 5.0 | 7.0 |
| Superior |  | 405 | 8217000 | 1.9 | 5.6 |
| Thunder |  | 41 | 1123 | 7.8 | 7.6 |
| Wollaston |  | 97 | 206200 | 3.0 | 7.4 |
| Cayuga |  | 125 | 17200 | — | 3.6 |
|  |  |  |  |  |  |
| CB153 ww | r | 0.25 | 0.44 | −0.02 | 0.01 |
|  | r^2^ | 0.06 | 0.20 | 0.00 | 0.00 |
| CB 52 ww | r | 0.20 | 0.59 | −0.09 | −0.29 |
|  | r^2^ | 0.04 | 0.35 | 0.01 | 0.08 |

Lake depth, area, DOC, and food web length (based on d15N) are from Houde et al. (2008b) and Guildford et al. (2008).

Abbreviations: DOC, dissolved organic carbon; TMF, trophic magnification factor; ww, wet weight.

**Table S4.** PBDE studies

| **Ecosystem** | **Geographic range** | **BDE congeners** | **End-point** | **Reference** |
| --- | --- | --- | --- | --- |
| FW lake | Temperate | 47, 99, 100, 209 | TMF | Law et al. (2006) |
| Marine | Arctic | 28, 47, 49, 66, 99, 100, 153, 154 | TMF | Kelly et al. (2008) |
| Marine | Temperate | 28, 47, 66, 99, 100, 153, 154, 183 | TMF | Wan et al. (2007) |
| FW reservoir | Sub-tropical | 28, 47, 66, 99, 100, 153, 154, 209 | TMF | Wu et al. (2009) |
| Marine | Subtropical | 28, 47, 66, 99, 100, 153, 154 | TMF | Yu et al. (2009) |
| Marine | Temperate | 28, 47, 49, 66, 85, 99, 100, 153, 154 | TMF | Losada et al. (2009) |
| FW lake | Temperate | 28, 47, 49, 66, 85, 99, 100, 153, 154, 183 | TMF | Hu et al. (2010) |
| FW lake | Temperate | 47, 99 | TMF | Borgå et al. (2012) |
| Marine | Temperate | 47 | TMF | Harmelin-Vivien et al. (2012) |
| FW lake | Temperate | 17, 28, 47, 66, 71, 85, 99, 100, 138, 153, 154, 183, 190, 209 | TMF | Yu et al. (2012) |
| Marine | Temperate | 28, 47, 66, 77, 99, 100, 153, 154 | TMF | Ma et al. (2013) |
| FW lake | Temperate | 28, 47, 99, 100, 153, 154, 183, 209 | TMF | Poma et al. (2014) |
| Marine | Temperate | 28, 47, 66, 99, 100, 153, 154, 183, 209 | TMF | Kobayashi et al. (2015) |
| FW lake | Temperate | 28, 47, 49+71, 66, 99, 100, 153, 154, 209 | TMF | Perez-Fuentetaja et al. (2015) |
| Marine | Temperate | 17, 28, 47, 66, 85, 99, 100, 138, 153, 154, 183, 209 | TMF | Shao et al. (2016) |
| FW lake | Temperate | 28, 47, 99, 100, 153, 154, 183 | TMF | Zhou et al. (2016) |
| Marine | Temperate | 47, 99, 100, 153, 154 | Reg | Mizukawa et al. (2009) |
| FW lake | Temperate | 47, 99, 100, 153, 154, 209 | Reg | Kuo et al. (2010) |

Studies explicitly aiming to determine TMFs or assessing PBDE biomagnification were searched on Scopus and Web of Science. The studies discussing biomagnification without isotopic ratio measurements were then discarded. Overall, 16 papers presented TMFs for a range of PBDEs (Table S4). Two studies assessed biomagnification by the mean of a regression of PBDE concentrations on δ15N without calculating TMFs and were also selected; conversely, a study on the Pearl River estuary, which applied a regression of PBDE concentrations on δ15N and found a negative slope (Guo et al. 2008), was discarded. Note that PBDEs were not necessarily the main focus in the papers recorded in Table 1 [e.g., the Lake Mjøsa study by Borgå et al. (2012) dealt with cyclic siloxanes and compared them with two PBDE congeners]. Abbreviations: BDE, bromodiphenylether; FW, freshwater; PBDE, polybromodiphenylether; TMF, trophic magnification factor

**Table S5.** TMF values for PFOS in aquatic food webs with fish as the top predator

| **Reference** | **TMF** | **Food web description and notes on each study** |
| --- | --- | --- |
| Martin et al. (2004) | 5.9 | Lake Ontario pelagic food web with lake trout (whole fish homogenate) as top predator. Observations on benthic organisms (one crustacean and one forage fish species) were excluded from the TMF calculation. TMF of 1.86 estimated for *Diporeia* to sculpin |
| Li et al. (2008) | 6.4 | Conducted in Gaobeidian Lake near outfall of a Beijing wastewater treatment plant. Species considered were zooplankton (whole) and four fish species (serum). TL from 2.0 (zooplankton) to 3.45 (crucian carp). TMF calculated by Franklin (2016) |
| Houde et al. (2008a) | 3.8 | Lake Ontario food web with lake trout (whole fish) as top predator. Benthic organisms (*Diporeia* and sculpin) included |
| Loi et al. (2011) | 1.3 | Subtropical brackish aquatic food web in Hong Kong (phytoplankton to top predator fish; whole-body homogenates) |
| Zhou et al. (2012) | ~1.0 | Freshwater food chain in Baiyangdian Lake, China. Reported concentrations were expressed on a dry-weight basis. Authors noted that sampling was “randomly with no standard food source” |
| Xu et al. (2014) | 2.9 | Sub-tropical freshwater aquatic and avian food web in Lake Taihu, China. Top predator bird (egret) included but TMF for fish would be the same given strong ln PFOS vs TL relationship |
| Fang et al. (2014) | 3.7 | Study conducted on a freshwater food chain in Lake Taihu, China. Based on fish from TL 2.5 to 4.24; invertebrates were excluded |
| Lescord et al. (2015) | ~0.7–1.0 | Based on benthic-based food webs of landlocked char in six arctic lakes (chironomids, juvenile char [whole fish], adult char [muscle]) |

Abbreviations: PFOS, perfluorooctane sulfonate; TL, trophic level; TMF, trophic magnification factor.

**Table S6.** Laboratory measured BCF values for dicofol

| **Species** | **Log BCF** | **Source** |
| --- | --- | --- |
| Carp | 3.84; 4.00 | National Institute of Technology and Evaluation (2018) |
| Hyalella azteca | 3.79; 4.04; 4.08 | Spehar et al. (1982) |
| Fathead minnow | 3.49; 3.63 | Spehar et al. (1982) |
|  | 4.02, 4.12, 4.14, 4.16, 4.18, 4.28 (day 28 values) | Eaton et al. (1983) |
| Zebrafish eleutheroembryos | 4.32 | El-Amrani et al. (2012) |
| Bluegill sunfish | 4.00 | US EPA (2009) |

Abbreviations: BCF, bioconcentration factor.

**Table S7.** Input parameters and conditions for the Arnot–Gobas AQUAWEBv1.2 model (Arnot and Gobas 2004)

|  | **Lipid (%)** | **NLOM (%)** | **%Moisture** | **NLOC** | **Weight (kg)** | **Estimated TL** |
| --- | --- | --- | --- | --- | --- | --- |
| Sediment | 0.00 | 0.00 | 97.00 | 3.00 | NA | NA |
| Phytoplankton | 0.50 | 0.00 | 93.00 | 6.50 | NA | 1.0 |
| Zooplankton | 1.20 | 20.00 | 78.80 | 0.00 | 5.70E-08 | 2.0 |
| Benthic invertebrates | 2.00 | 20.00 | 78.00 | 0.00 | 1.00E-05 | 2.0 |
| Forage fish | 3.00 | 20.00 | 77.00 | 0.00 | 0.01 | 3.0 |
| Piscivorous fish | 3.00 | 20.00 | 77.00 | 0.00 | 0.01 | 4.0 |
|  |  |  |  |  |  |  |
| Temperature |  |  | 15°C |  |  |  |
| Sediment-water column disequilibrium (π_socw_/K_ow_) | | | 5 |  |  |  |
| Concentration of chemical in water column (C_W_) | | | 1 ng/L |  |  |  |
| Concentration of chemical in sediment pore water (ng/L) | | | = C_sed_/(K_OW_ × 0.35 × *f*_OC_) | |  |  |
| Concentration of chemical in sediment (C_sed_) (ng/kg dw) | | | = 5 × K_OW_ × C_W_ × 3.0% | |  |  |
| Diet for benthic invertebrates | | | 100% sediment | |  |  |
| Benthic invertebrate fraction pore water (fpw) | | | 5% |  |  |  |
| Diet for zooplankton | | | 100% phytoplankton | |  |  |
| Diet for forage fish | | | 30% benthic invertebrates  70% zooplankton | |  |  |
| Diet for piscivorus fish | | | 100% forage fish | |  |  |
| Water density (kg/L) |  |  | 1.0 |  |  |  |
| Lipid density (kg/L) |  |  | 0.9 |  |  |  |

Abbreviations: NA, not applicable; TL, trophic level.

**Table S8.** TMF values commonly reported for PAHs identified as priority substance/priority hazardous substance under the WFD and main attributes of aquatic food webs from which they are derived.

| **Chemical compound** | **Latitudinal class** | **Type of ecosystem** | **Type of food web** | **Species composition** | **TL range** | **FWMF/**  **TMF** | **Fish tissue/organ for chemical measurements** | **Source** |
| --- | --- | --- | --- | --- | --- | --- | --- | --- |
| B[a]P | Temperate | Brackish | Pelagic | PP, ZP, macro-invertebrates, fish | 1.0–3.98 | 0.38 | Muscle, head, and intestinal tract (emptied and rinsed) | Nfon et al. (2008) |
| B[a]P | Sub-tropical | Freshwater (lake) | Benthic-pelagic | Fish only | 1.06–3.47 | 1.47 | Muscle tissue | Wang et al. (2012) |
| B[a]P | Temperate | Marine (coastal) | Benthic-pelagic | PP, ZP, macro-invertebrates, fish, seabirds | 1.6–3.88 | 0.32* | Muscle tissue | Wan et al. (2007) |
| B[a]P | Temperate | Fresh-brackish (estuary) | Benthic-pelagic | Shellfish, fish | 2.0–4.0 | 0.07* | Skinless fish, head, guts and bones discarded | Khairy et al. (2014) |
| B[a]P | Temperate | Estuary | Benthic-pelagic | SPM, PP, ZP, macro-invertebrates, fish, seabirds | 1.0–4.9 | 0.32* (individual),  0.12* (geo mean) | Whole fish | Brisebois (2013) |
| B[a]P | Sub-tropical | Marine (coastal) | Benthic-pelagic | Macroinvertebrates, fish |  | 0.47 | Muscle tissue | Takeuchi et al. (2009) |
| Fluoranthene | Temperate | Brackish | Pelagic | PP, ZP, macro-invertebrates, fish | 1.0–3.98 | 0.33* | Muscle, head, and intestinal tract (emptied and rinsed) | Nfon et al. (2008) |
| Fluoranthene | Sub-tropical | Freshwater (lake) | Benthic-pelagic | Fish only | 1.06–3.47 | 1.20 | Muscle tissue | Wang et al. (2012) |
| Fluoranthene | Temperate | Marine (coastal) | Benthic-pelagic | PP, ZP, macro-invertebrates, fish, seabirds | 1.6–3.88 | 0.11* | Muscle tissue | Wan et al. (2007) |
| Fluoranthene | Temperate | Fresh-brackish (estuary) | Benthic-pelagic | shellfish, fish | 2.0–4.0 | 0.26* | Skinless fish, head, guts and bones removed | Khairy et al. (2014) |
| Fluoranthene | Sub-tropical | Marine (coastal) | Benthic-pelagic | Macroinvertebrates, fish |  | 0.17* | Muscle tissue | Takeuchi et al. (2009) |

TMF values from Nfon et al. (2008) and Takeuchi et al. (2009) were recalculated from the slopes (*m*) of the linear regressions between the ln (or log_10_) of chemical concentrations and δ^15^N (where δ^15^N is used as a measure of the relative trophic level), using the following equation: TMF = 10^(^*^m^*^×3.4)^ or *e*^(^*^m^*^×3.4)^.

Abbreviations: B[a]P, benzo(a)pyrene; FWMF, food web magnification factor; PAH, polyaromatic hydrocarbons; PP, phytoplankton; SPM, suspended particulate matter; TL, trophic level; TMF, trophic magnification factor; WFD, Water Framework Directive; ZP, zooplankton.

*These TMF values represent a statistically significant increase or decrease in lipid-normalized concentrations with increasing trophic level.

**Supporting Information References**

Arnot JA, Gobas FA. 2004. A food web bioaccumulation model for organic chemicals in aquatic ecosystems. *Environ Toxicol Chem* **23**:2343–2355.

Borgå K, Fjeld E, Kierkegaard A, McLachlan M. 2012. Food web accumulation of cyclic siloxanes in Lake Mjøsa, Norway. *Environ Sci Technol* **46**:6347–6354.

Brisebois A. 2013. Relationship between the bioconcentration factor (BCF), the bioaccumulation factor (BAF), and the trophic magnification factor (TMF) [Master's thesis]. Burnaby (BC): Simon Fraser University.

Clayden MG, Kidd KA, Wyn B, Kirk JL, Muir DC, O'Driscoll NJ. 2013. Mercury biomagnification through food webs is affected by physical and chemical characteristics of lakes. *Environ Sci Technol* **47**:12047–12053.

Eaton JG, Mattson VR, Mueller LH, Tanner DK. 1983. Effects of suspended clay on bioconcentration of Kelthane in fathead minnows. *Arch Environ Contam Toxicol* **12**:439–445.

El-Amrani S, Pena-Abaurrea M, Sanz-Landaluze J, Ramos L, Guinea J, Camara C. 2012. Bioconcentration of pesticides in zebrafish eleutheroembryos (Danio rerio). *Sci Total Environ* **425**:184–190.

Fang S, Chen X, Zhao S, Zhang Y, Jiang W, Yang L, Zhu L. 2014. Trophic magnification and isomer fractionation of perfluoroalkyl substances in the food web of Taihu Lake, China. *Environ Sci Technol* **48**:2173–2182.

Franklin J. 2016. How reliable are field-derived biomagnification factors and trophic magnification factors as indicators of bioaccumulation potential? Conclusions from a case study on per- and polyfluoroalkyl substances. *Integr Environ Assess Manage* **12**:6–20.

Guildford SJ, Muir DC, Houde M, Evans MS, Kidd KA, Whittle DM, Drouillard K, Wang X, Anderson MR, Bronte CR, Devault DS, Haffner D, Payne J, Kling HJ. 2008. PCB concentrations in lake trout (Salvelinus namaycush) are correlated to habitat use and lake characteristics. *Environ Sci Technol* **42**:8239-8244.

Guo X, Cai W-J, Zhai W, Dai M, Wang Y, Chen B. 2008. Seasonal variations in the inorganic carbon system in the Pearl River (Zhujiang) estuary. *Cont Shelf Res* **28**:1424–1434.

Harmelin-Vivien M, Bodiguel X, Charmasson S, Loizeau V, Mellon-Duval C, Tronczyński J, Cossa D. 2012. Differential biomagnification of PCB, PBDE, Hg and Radiocesium in the food web of the European hake from the NW Mediterranean. *Mar Pollut Bull* **64**:974–983.

Houde M, Czub G, Small JM, Backus S, Wang X, Alaee M, Muir DCG. 2008a. Fractionation and bioaccumulation of perfluorooctane sulfonate (PFOS) isomers in a Lake Ontario food web. *Environ Sci Technol* **42**:9397–9403.

Houde M, Muir DCG, Kidd KA, Guildford S, Drouillard K, Evans MS, Wang X, Whittle DM, Haffner D, Kling H. 2008b. Influence of lake characteristics on the biomagnification of persistent organic pollutants in lake trout food webs. *Environ Toxicol Chem* **27**:2169–2178.

Hu G-c, Dai J-y, Xu Z-c, Luo X-j, Cao H, Wang J-s, Mai B-x, Xu M-q. 2010. Bioaccumulation behavior of polybrominated diphenyl ethers (PBDEs) in the freshwater food chain of Baiyangdian Lake, North China. *Environ Int* **36**:309–315.

Kelly BC, Ikonomou MG, Blair JD, Gobas FAPC. 2008. Bioaccumulation behaviour of polybrominated diphenyl ethers (PBDEs) in a Canadian Arctic marine food web. *Sci Total Environ* **401**:60–72.

Khairy MA, Weinstein MP, Lohmann R. 2014. Trophodynamic behavior of hydrophobic organic contaminants in the aquatic food web of a tidal river. *Environ Sci Technol* **48**:12533–12542.

Kobayashi J, Imuta Y, Komorita T, Yamada K, Ishibashi H, Ishihara F, Nakashima N, Sakai J, Arizono K, Koga M. 2015. Trophic magnification of polychlorinated biphenyls and polybrominated diphenyl ethers in an estuarine food web of the Ariake Sea, Japan. *Chemosphere* **118**:201–206.

Kuo Y-M, Sepúlveda M, Hua I, Ochoa-Acuña H, Sutton T. 2010. Bioaccumulation and biomagnification of polybrominated diphenyl ethers in a food web of Lake Michigan. *Ecotoxicology* **19**:623–634.

Law K, Halldorson T, Danell R, Stern G, Gewurtz S, Alaee M, Marvin C, Whittle M, Tomy G. 2006. Bioaccumulation and trophic transfer of some brominated flame retardants in a Lake Winnipeg (Canada) food web. *Environ Toxicol Chem* **25**:2177–2186.

Lescord GL, Kidd KA, De Silva AO, Williamson M, Spencer C, Wang X, Muir DCG. 2015. Perfluorinated and polyfluorinated compounds in lake food webs from the Canadian High Arctic. *Environ Sci Technol* **49**:2694–2702.

Li X, Yin Yeung LW, Xu M, Taniyasu S, Lam PKS, Yamashita N, Dai J. 2008. Perfluorooctane sulfonate (PFOS) and other fluorochemicals in fish blood collected near the outfall of wastewater treatment plant (WWTP) in Beijing. *Environ Pollut* **156**:1298–1303.

Loi EIH, Yeung LWY, Taniyasu S, Lam PKS, Kannan K, Yamashita N. 2011. Trophic magnification of poly- and perfluorinated compounds in a subtropical food web. *Environ Sci Technol* **45**:5506–5513.

Losada S, Roach A, Roosens L, Santos FJ, Galceran MT, Vetter W, Neels H, Covaci A. 2009. Biomagnification of anthropogenic and naturally-produced organobrominated compounds in a marine food web from Sydney Harbour, Australia. *Environ Int* **35**:1142–1149.

Ma X, Zhang H, Yao Z, Zhao X, Wang L, Wang Z, Chen J, Chen J. 2013. Bioaccumulation and trophic transfer of polybrominated diphenyl ethers (PBDEs) in a marine food web from Liaodong Bay, North China. *Mar Pollut Bull* **74**:110–115.

Martin JW, Whittle DM, Muir DCG, Mabury SA. 2004. Perfluoroalkyl contaminants in a food web from Lake Ontario. *Environ Sci Technol* **38**:5379–5385.

Mizukawa K, Takada H, Takeuchi I, Ikemoto T, Omori K, Tsuchiya K. 2009. Bioconcentration and biomagnification of polybrominated diphenyl ethers (PBDEs) through lower-trophic-level coastal marine food web. *Mar Pollut Bull* **58**:1217–1224.

National Institute of Technology and Evaluation. 2018. Chemical Management [Internet]. [cited 2018 Mar 4]. Available from: http://www.nite.go.jp/en/chem/qsar/cscl_data.html

Nfon E, Cousins IT, Broman D. 2008. Biomagnification of organic pollutants in benthic and pelagic marine food chains from the Baltic Sea. *Sci Total Environ* **397**:190–204.

Perez-Fuentetaja A, Mackintosh SA, Zimmerman LR, Clapsadl MD, Alaee M, Aga DS. 2015. Trophic transfer of flame retardants (PBDEs) in the food web of Lake Erie. *Can J Fish Aquat Sci* **72**:1886–1896.

Poma G, Volta P, Roscioli C, Bettinetti R, Guzzella L. 2014. Concentrations and trophic interactions of novel brominated flame retardants, HBCD, and PBDEs in zooplankton and fish from Lake Maggiore (Northern Italy). *Science of the Total Environment* **481**:401-408.

Shao M, Tao P, Wang M, Jia H, Li Y-F. 2016. Trophic magnification of polybrominated diphenyl ethers in the marine food web from coastal area of Bohai Bay, North China. *Environ Pollut* **213**:379–385.

Spehar RL, Tanner DK, Gibson JH. 1982. Effects of kelthane and pydrin on early life stages of fathead minnows (Pimephales promelas) and amphipods (Hyalella azteca). In: *Aquatic Toxicology and Hazard Assessment*. West Conshohocken (PA): ASTM International, pp. 234–245.

Takeuchi I, Miyoshi N, Mizukawa K, Takada H, Ikemoto T, Omori K, Tsuchiya K. 2009. Biomagnification profiles of polycyclic aromatic hydrocarbons, alkylphenols and polychlorinated biphenyls in Tokyo Bay elucidated by δ13C and δ15N isotope ratios as guides to trophic web structure. *Mar Pollut Bull* **58**:663–671.

US EPA. 2009. *Risks of Dicofol Use to Federally Threatened California Red-Legged Frog (Rana aurora draytonii): Pesticide Effects Determination*. Washington (DC): Environmental Fate and Effects Division, Office of Pesticide Programs.

Wan Y, Jin X, Hu J, Jin F. 2007. Trophic dilution of polycyclic aromatic hydrocarbons (PAHs) in a marine food web from Bohai Bay, North China. *Environ Sci Technol* **41**:3109–3114.

Wang D-Q, Yu Y-X, Zhang X-Y, Zhang S-H, Pang Y-P, Zhang X-L, Yu Z-Q, Wu M-H, Fu J-M. 2012. Polycyclic aromatic hydrocarbons and organochlorine pesticides in fish from Taihu Lake: their levels, sources, and biomagnification. *Ecotoxicol Environ Saf* **82**:63–70.

Wu JP, Luo XJ, Zhang Y, Yu M, Chen SJ, Mai BX, Yang ZY. 2009. Biomagnification of polybrominated diphenyl ethers (PBDEs) and polychlorinated biphenyls in a highly contaminated freshwater food web from South China. *Environ Pollut* **157**:904–909.

Wyn B, Kidd KA, Burgess NM, Curry RA. 2009. Mercury biomagnification in the food webs of acidic lakes in Kejimkujik National Park and National Historic Site, Nova Scotia. *Can J Fish Aquat Sci* **66**:1532–1545.

Xu J, Guo CS, Zhang Y, Meng W. 2014. Bioaccumulation and trophic transfer of perfluorinated compounds in a eutrophic freshwater food web. *Environ Pollut* **184**:254–261.

Yu M, Luo X-J, Wu J-P, Chen S-J, Mai B-X. 2009. Bioaccumulation and trophic transfer of polybrominated diphenyl ethers (PBDEs) in biota from the Pearl River Estuary, South China. *Environ Int* **35**:1090–1095.

Yu Y-X, Zhang S-H, Huang N-B, Li J-L, Pang Y-P, Zhang X-Y, Yu Z-Q, Xu Z-G. 2012. Polybrominated diphenyl ethers and polychlorinated biphenyls in freshwater fish from Taihu Lake, China: Their levels, biomagnification, and its influencing factors. *Environ Toxicol Chem* **31**:542–549.

Zhou YH, Chen QF, Du XY, Yin G, Qiu YL, Ye L, Zhu ZL, Zhao JF. 2016. Occurrence and trophic magnification of polybrominated diphenyl ethers (PBDEs) and their methoxylated derivatives in freshwater fish from Dianshan Lake, Shanghai, China. *Environ Pollut* **219**:932–938.

Zhou Z, Shi Y, Li W, Xu L, Cai Y. 2012. Perfluorinated compounds in surface water and organisms from Baiyangdian Lake in North China: source profiles, bioaccumulation and potential risk. *Bull Environ Contam Toxicol* **89**:519–524.
